# Impact of sex disparities on the clinical manifestations in patients with systemic lupus erythematosus: A systematic review and meta-analysis

**DOI:** 10.1097/MD.0000000000004272

**Published:** 2016-07-22

**Authors:** Kamini Devi Boodhoo, Sijia Liu, Xiaoxia Zuo

**Affiliations:** Department of Rheumatology, Xiangya Hospital, Central South University, Changsha, Hunan, People's Republic of China.

**Keywords:** clinical manifestations, meta-analysis, sex differences, systemic lupus erythematosus

## Abstract

**Background:**

Systemic lupus erythematosus (SLE) is a chronic autoimmune multiorgan disorder of unknown etiology. It affects both men and women, but with different disease manifestations of differing disease severity and in varying proportion, with a female predominance of approximately 90%. There have been numerous studies addressing this issue, especially its implications in relation to optimal sex-tailored treatment and improvement of survival rate; however, further research is warranted. A meta-analysis of studies was performed to compare the impact of sex on the clinical outcomes of SLE in different populations.

**Methods:**

A literature search of the MEDLINE/PubMed and EMBASE databases (until January 2016) was conducted to identify relevant articles. Clinical manifestations reported in these patients were considered as endpoints for this meta-analysis. Two independent reviewers determined eligibility criteria. A fixed-effect model has been used where a small heterogeneity was observed, or else, a random-effect model has been used among the studies. Odd ratio (OR) with 95% confidence interval (CI) was used to express the pooled effect on dichotomous variables, and the pooled analyses were performed with RevMan 5.3.

**Results:**

Sixteen studies consisting of a total of 11,934 SLE patients (10,331 females and 1603 males) have been included in this meta-analysis. The average female-to-male ratio of all the included studies is around 9.3:1. Several statistically significant differences were found: alopecia, photosensitivity, and oral ulcers were significantly higher in female patients (OR 0.36, 95% CI 0.29–0.46, *P* < 0.00001; OR 0.72, 95% CI 0.63–0.83, *P* < 0.00001; and OR 0.70, 95% CI 0.60–0.82, *P* < 0.00001, respectively). Malar rash was significantly higher in female patients (OR 0.68, 95% CI 0.53–0.88, *P* = 0.003), and arthritis was significantly lower in male patients (OR 0.72, 95% CI 1.25–1.84, *P* < 0.00001). However, serositis and pleurisies were significantly higher in female patients (OR 1.52, 95% CI 1.25–1.84 *P* < 0.0001; and OR 1.26, 95% CI 1.07–1.48, *P* = 0.006, respectively). Renal involvement was higher in male patients (OR 1.51, 95% CI 1.31–1.75, *P* < 0.00001).

**Conclusion:**

The results of this meta-analysis suggest that alopecia, photosensitivity, oral ulcers, arthritis, malar rash, lupus anticoagulant level, and low level of C3 were significantly higher in female lupus patients, whereas renal involvement, serositis and pleurisies, thrombocytopenia, and anti-double stranded deoxyribonucleic acid level were predominant in male patients.

## 1 Introduction

Systemic lupus erythematosus (SLE) is a chronic inflammatory disease of unknown etiology involving multiple organ systems. It occurs after the loss of self-tolerance of the immune system, which leads to the development of autoantibodies against nuclear antigens, immune complex formation, inflammation, and eventually permanent organ injury. It affects predominantly women, primarily during the reproductive age, with a lower ratio seen before puberty and a decline later in life. The incidence of SLE varies according to the characteristics of each population, such as patients’ age, sex, and ethnicity. Sex differences may influence the clinical and serological expression, therapy, and outcome. Epidemiologic studies report the occurrence of SLE varies among different countries and different ethnic groups.^[1,2]^ These differences suggest that besides hormonal and genetic susceptibility, geographic and environmental factors are also implicated in the development of this connective tissue disease.^[1,2]^ Whereas SLE is more common in women than in men, male patients are thought to have more severe disease than females.^[3]^ Over 5-year follow-up, Stefanidou et al^[4]^ found that male sex might be a poor factor in SLE prognosis.

The objectives of this study were to conduct a systemic literature review and meta-analysis of studies that directly compared the difference in clinical outcomes between male and female lupus patients in various population groups.

## 2 Methods

### 2.1 Data sources and search strategy

Medline and EMBASE were searched for studies comparing the clinical manifestations in male and female SLE patients by typing the words/phrases “systemic lupus erythematosus and gender differences.” To further enhance this search, the abbreviations “SLE” and the words “sex disparities” have also been used. Reference lists were also searched for relevant titles. Official Web sites of certain journals such as “Medicine” have also been searched for relevant articles.

### 2.2 Study selection

#### 2.2.1 Inclusion and exclusion criteria

Studies were included if:

- They compared the clinical manifestations in male and female SLE patients.
- Their data were available for comparison (including data for both the experimental and control groups).
- Full text articles were available.

Studies were excluded if:

- They were case studies, letter to editors, or review articles.
- Clinical manifestations were not reported as their endpoints.
- Full text articles were not available.
- Duplicates.

### 2.3 Outcomes

Outcomes analyzed in this meta-analysis included the following:

1. Clinical manifestations of the
  - Cardiovascular system
  - Respiratory system
  - Renal system
  - Connective tissues system
  - Hematological system
  - Dermatological system
  - Neurological system
2. Manifestations of certain organ systems according to the Systemic Lupus International Collaborating Clinics/American College of Rheumatology Damage Index:
  - Cardiovascular: pericarditis
  - Lungs: pleurisies
  - Skin: alopecia, malar rash, discoid rash, photosensitivity
  - Blood: hematological involvement, hemolytic anemia, leukopenia, lymphopenia, thrombocytopenia
  - Connective tissues: arthritis
  - Neurological: neurological involvement, seizures, psychosis
  - Renal: lupus nephritis

The reported outcomes of the included studies have been represented in Tables 1–4.

**Table 1:**
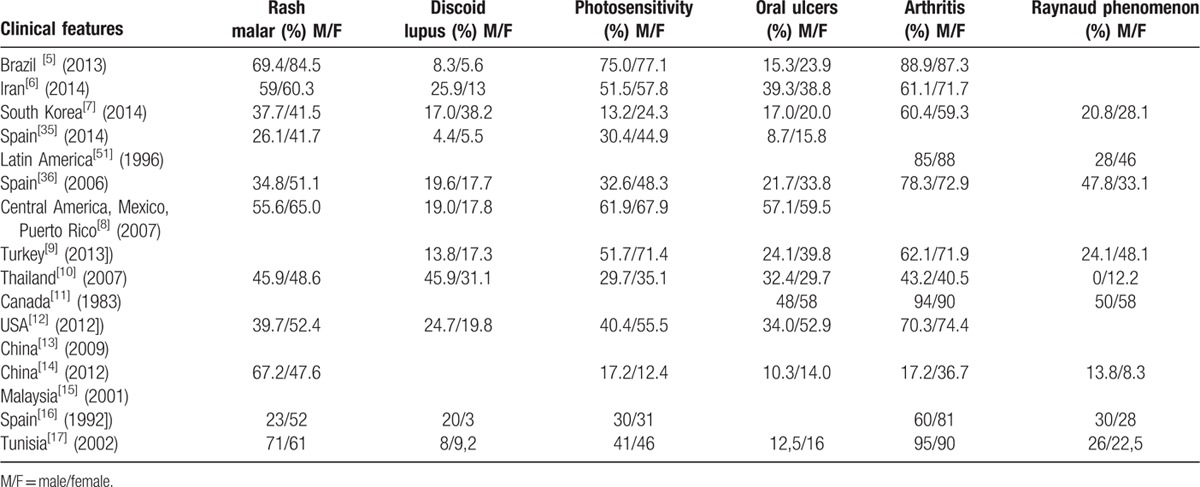
Demographical and clinical manifestations of male and female lupus patients.

**Table 2:**
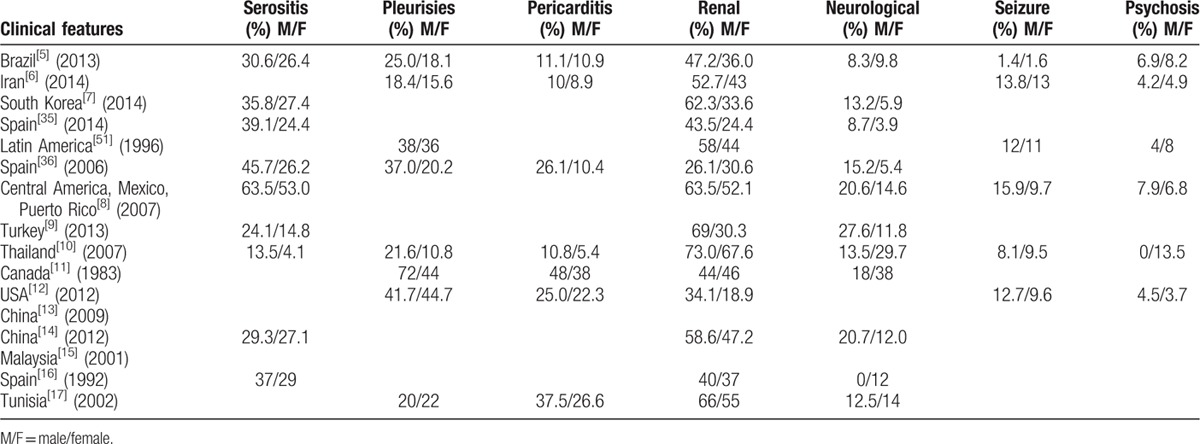
Demographical and clinical manifestations of male and female lupus patients (continued).

**Table 3:**
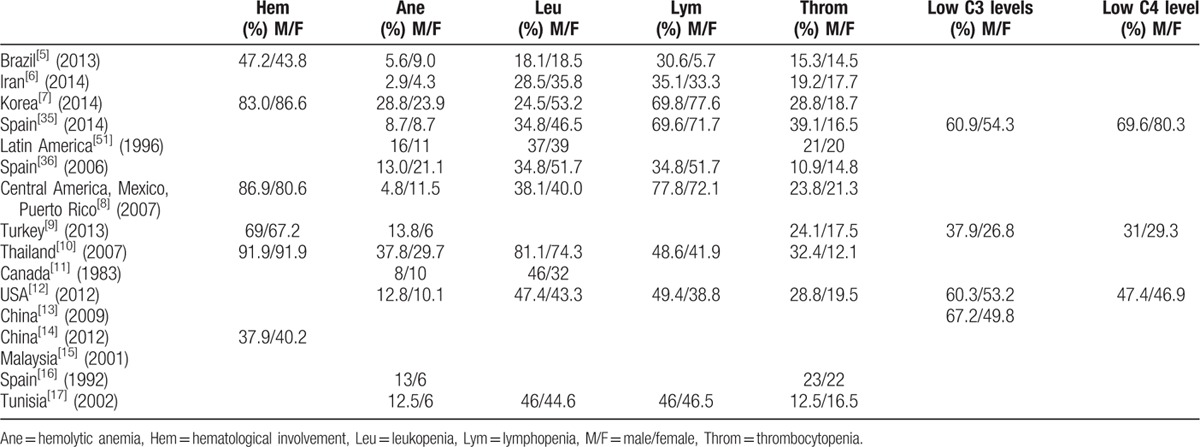
Hematological profile and complement levels of male and female lupus patients.

**Table 4:**
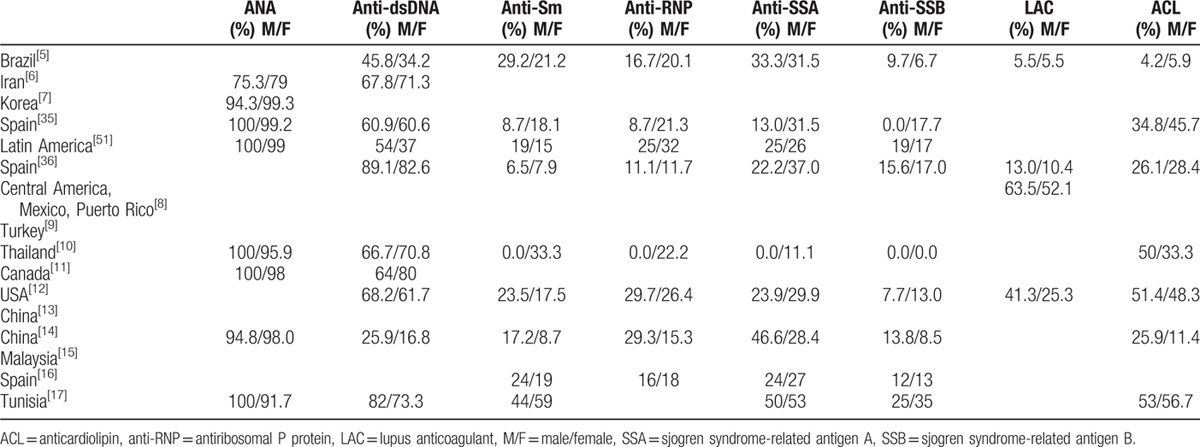
The autoantibody positivity of male and female lupus patients.

### 2.4 Data extraction and quality assessment

Two authors (KDB and SL) independently reviewed the data and assessed the eligibility and methodological quality of each eligible study. Information regarding type and length of study, location and number of patients, clinical manifestations, and authors’ first names were systematically extracted. Disagreements were discussed between the authors, and if the authors could not reach a consensus, disagreements were resolved by the third author (XZ). The bias risk within the studies was assessed with the components recommended by the Cochrane Collaboration.^[18]^

### 2.5 Methodological quality and statistical analysis

Heterogeneity across trials was assessed using the Cochrane *Q*-statistic (*P* ≤ 0.05 was considered significant) and *I*^2^-statistic. *I*^2^ described the percentage of total variation across studies, which is due to heterogeneity rather than chance. A value of 0% indicated no heterogeneity, and larger values indicated increase heterogeneity. If *I*^2^ was <50%, fixed-effect model was used. However, if *I*^2^ was >50%, a random-effect model was used. Publication bias was visually estimated by assessing funnel plots. We calculated odd ratios (ORs) and 95% confidence intervals (CIs) for categorical variables. The pooled analyses were performed with RevMan 5.3 software. The authors had full access to and take full responsibility for the integrity of the data. All authors have read and agreed to the manuscript as written.

### 2.6 Ethics

Ethical approval was not necessary as this study is a “Systematic Review and Meta-analysis.”

## 3 Results

### 3.1 Search results

**Figure 1:**
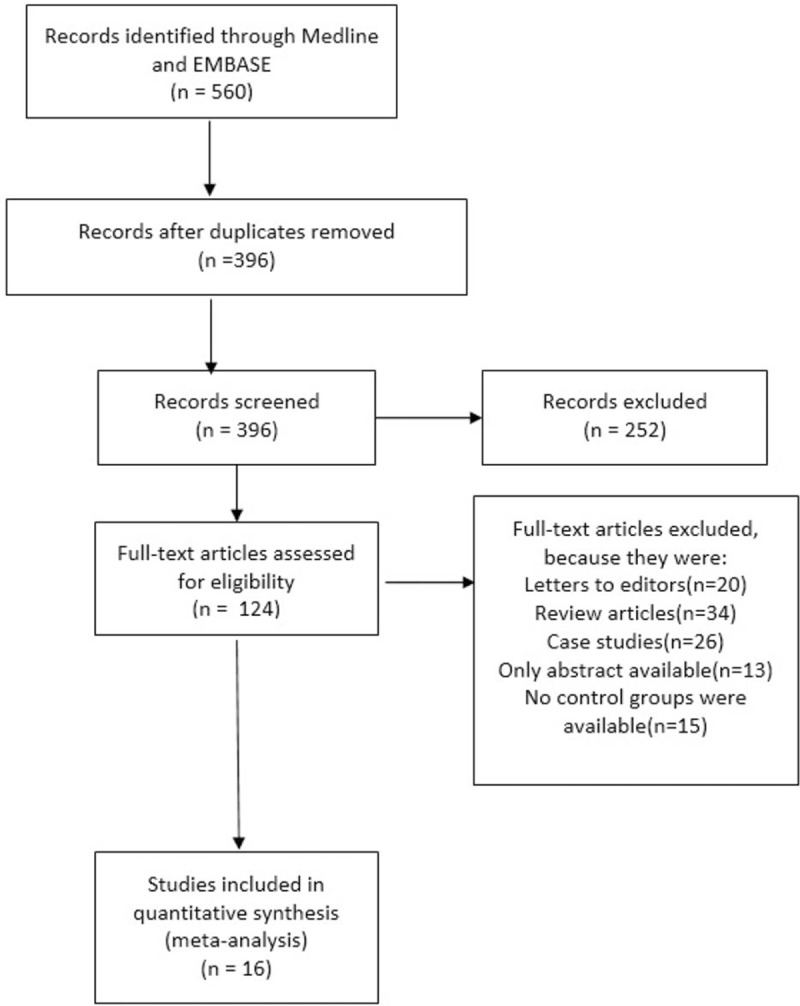
Flow diagram of the study selection.

Study selection, data collection, analysis, and reporting of the results were performed using the recommendations of the Preferred Reporting Items for Systematic Reviews and Meta-Analyses (PRISMA) statement.^[19]^ A total of 560 articles were obtained during the search process. Among them, 396 articles were eliminated because they were either duplicates or they were not related to our topic. The remaining 124 full-text articles were assessed for eligibility. A further 95 articles were eliminated because they were letter to editors, review articles, or case studies. Among the 29 remaining articles, 13 more studies were eliminated because either only their abstract parts were available, or there were no control groups for comparison. After strictly considering the inclusion and exclusion criteria, 16 articles were finally selected for this systematic review and meta-analysis. The study selection including the flow of the process for identifying potentially eligible trials has been represented in Fig. 1. The characteristics of the 16 studies that met the eligibility criteria are displayed in Tables 5 and 6.

**Table 5:**
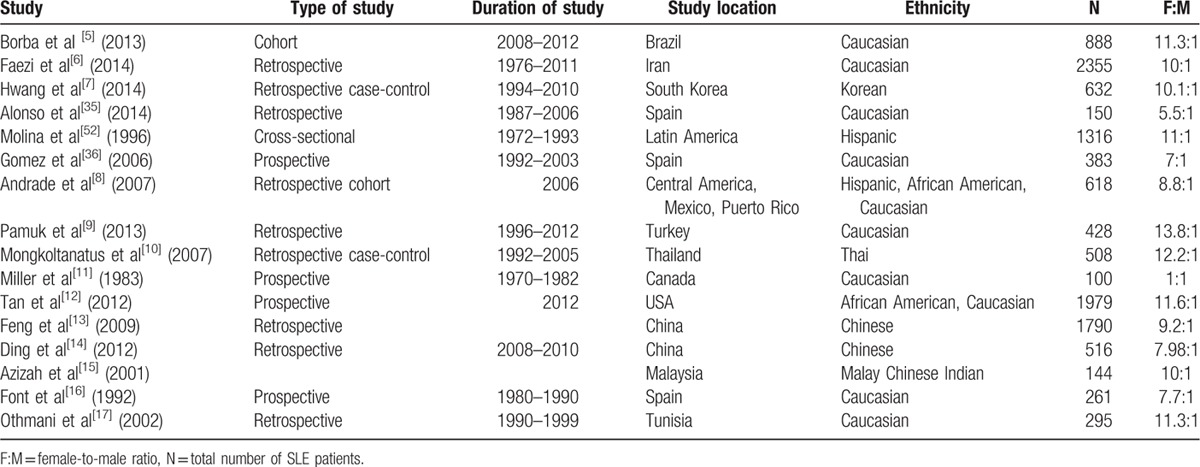
General characteristics of the included studies.

**Table 6:**
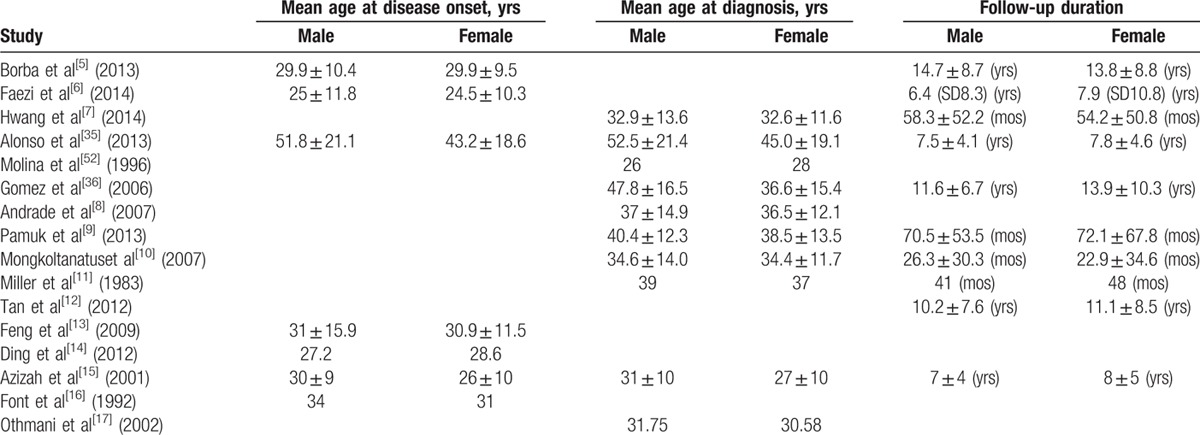
General characteristics of the included studies.

### 3.2 Description of the included studies

The 16 articles included in the meta-analysis incorporated a total of 11934 lupus patients, with 1603 males and 10331 females from many different locations such as America, Latin America, Spain, China, Malaysia, Iran, Turkey, Korea, Taiwan, Canada, and Brazil. Baseline characteristics of the studies, including sample size, type and duration of study, study location, ethnicity, female-to-male ratio, mean age at time of diagnosis, mean age at disease onset, and length of follow-up are outlined in Tables 5 and 6.

### 3.3 Results of our analysis

**Figure 2:**
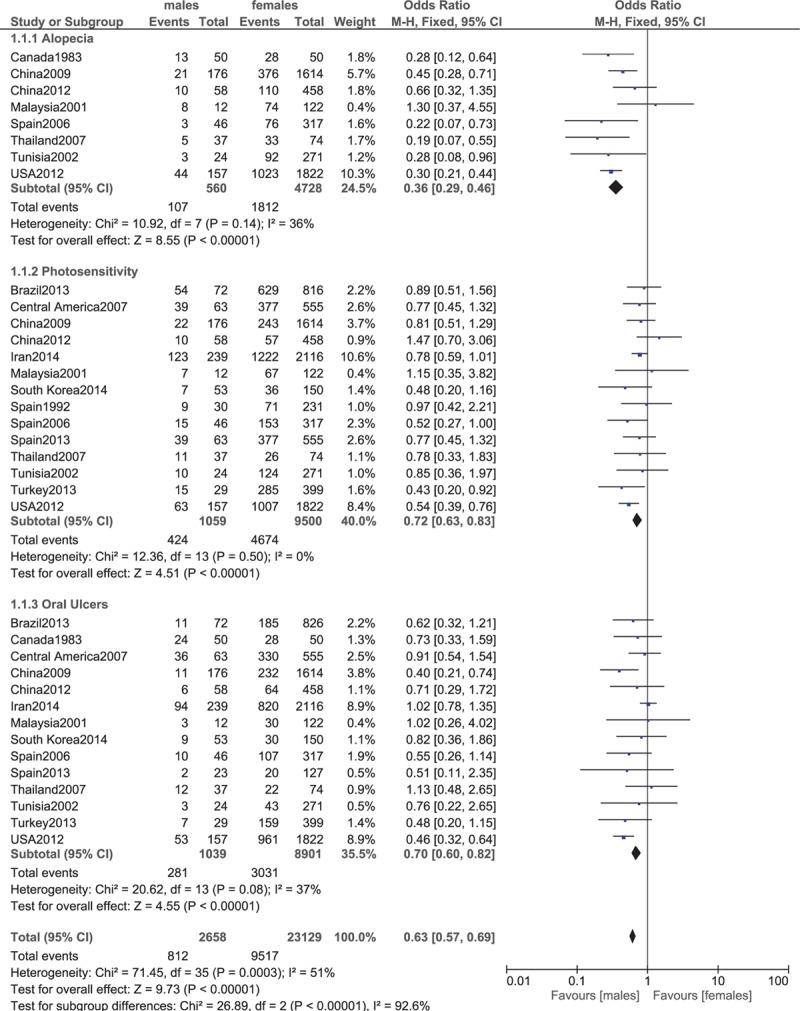
Alopecia, photosensitivity, oral ulcers.

**Figure 3:**
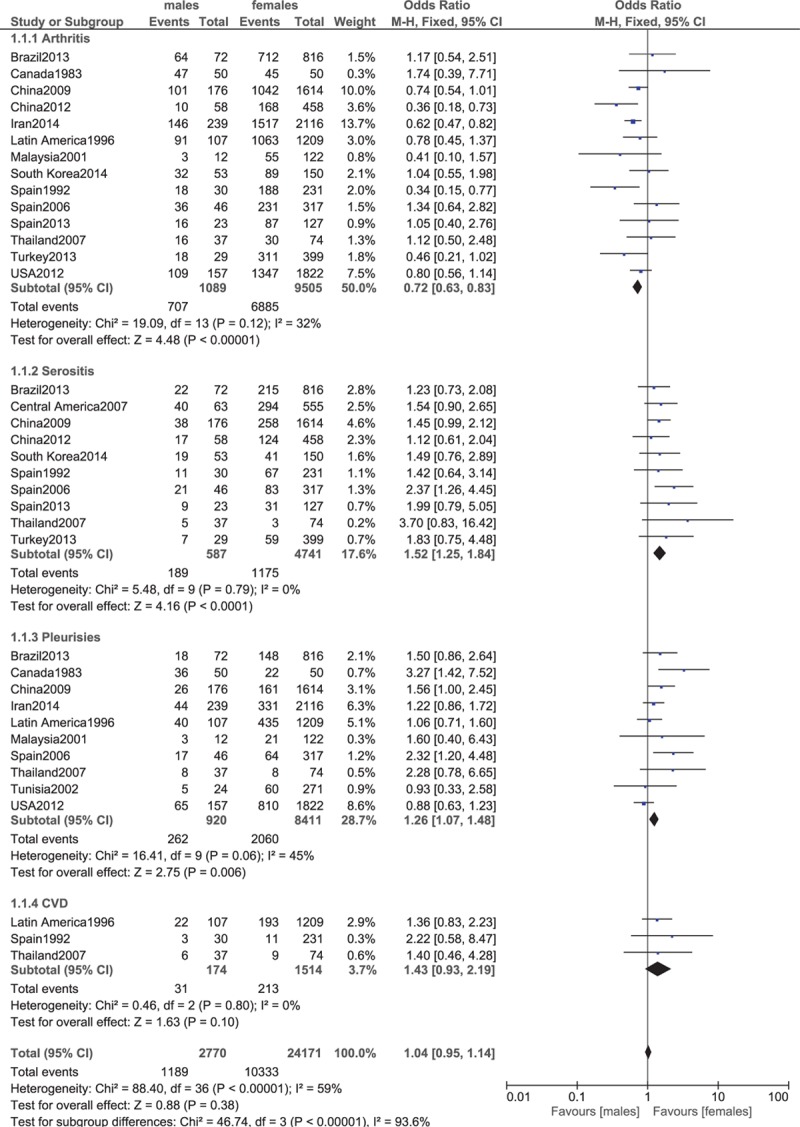
Arthritis, serositis, pleurisies, cardiovascular disease (CVD).

**Figure 4:**
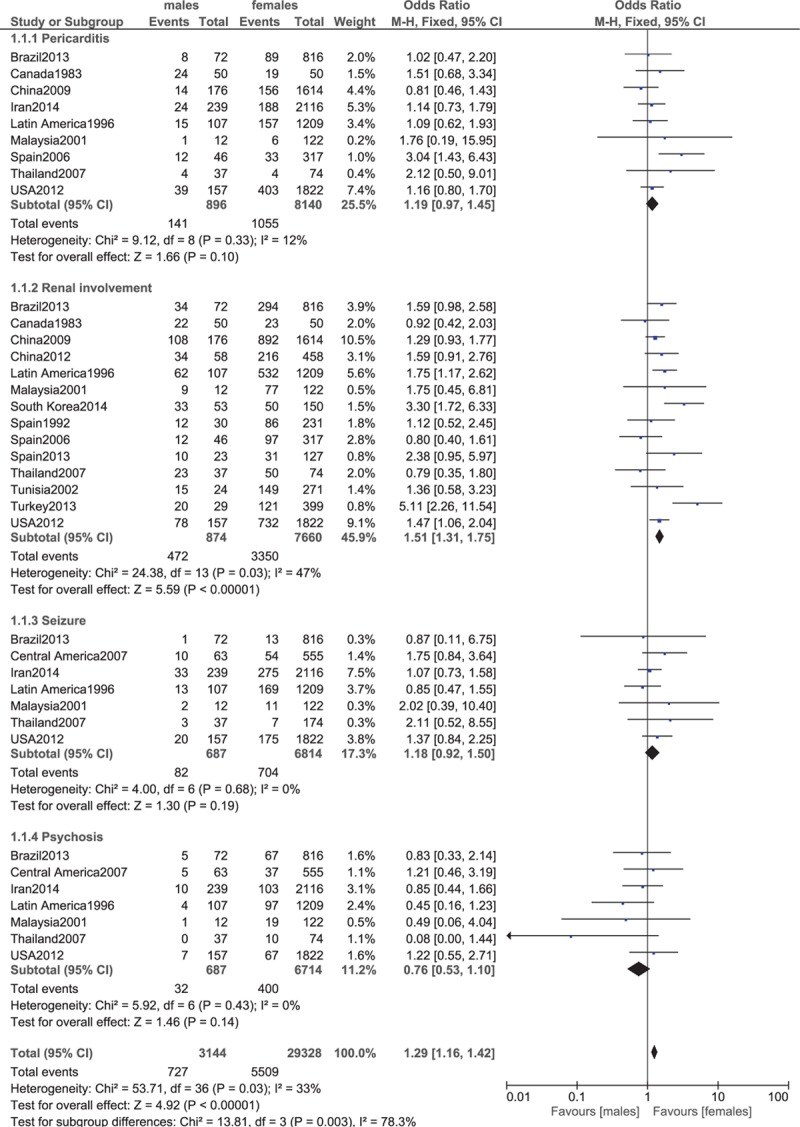
Pericarditis, renal involvement, seizure, psychosis.

**Figure 5:**
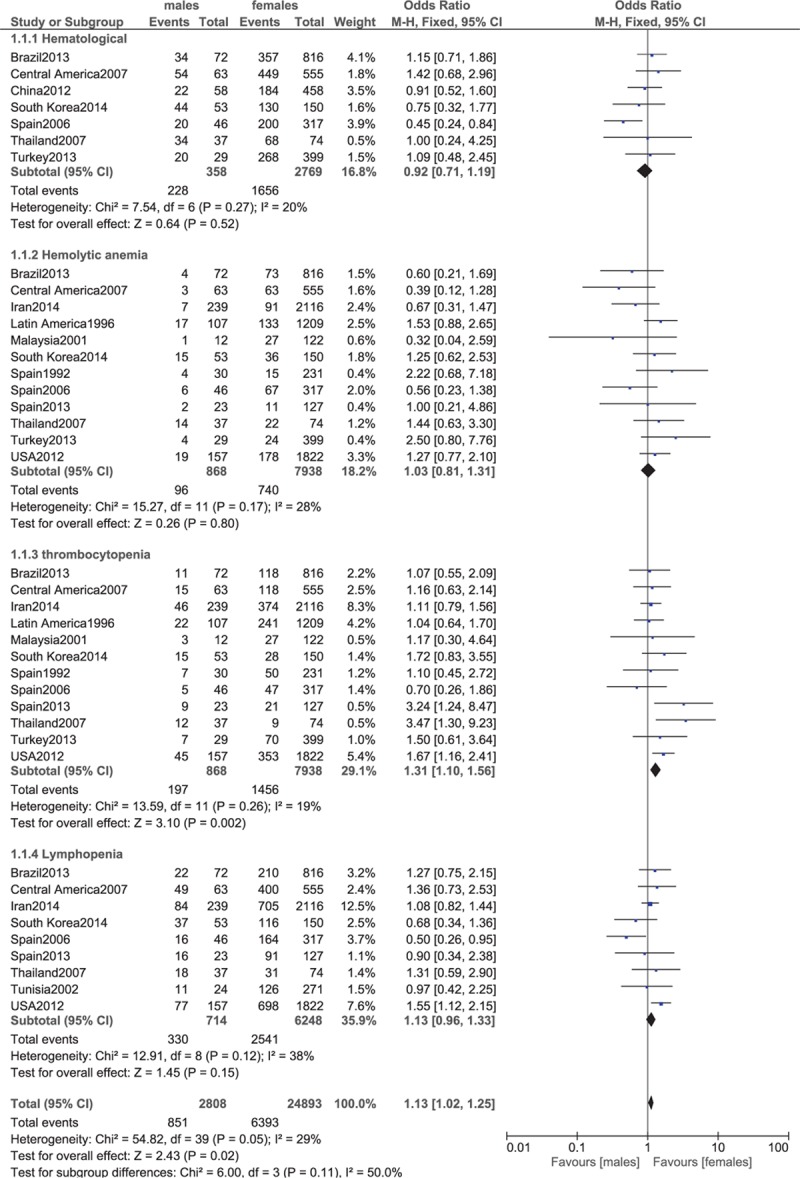
Hematological manifestations, hemolytic anemia, lymphopenia.

**Figure 6:**
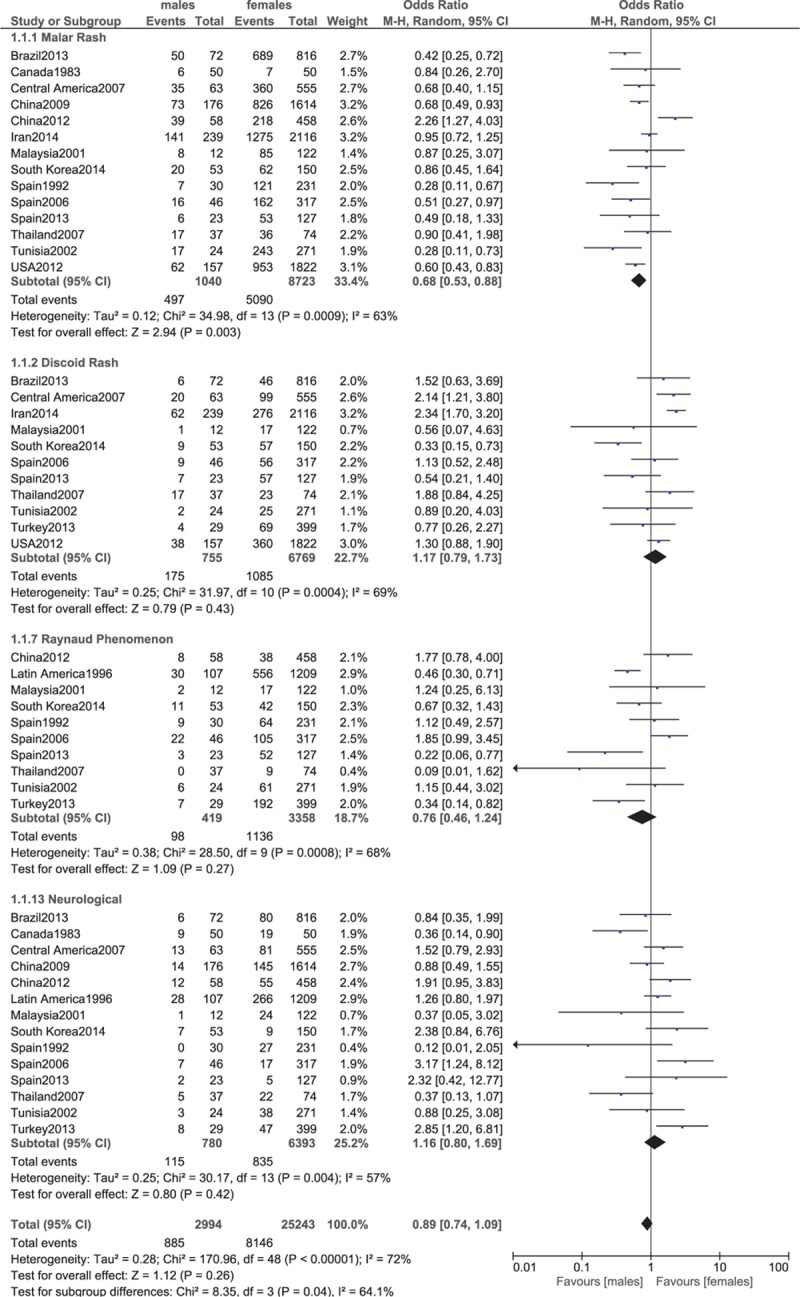
Malar rash, discoid rash, Raynaud phenomenon, neurological.

**Figure 7:**
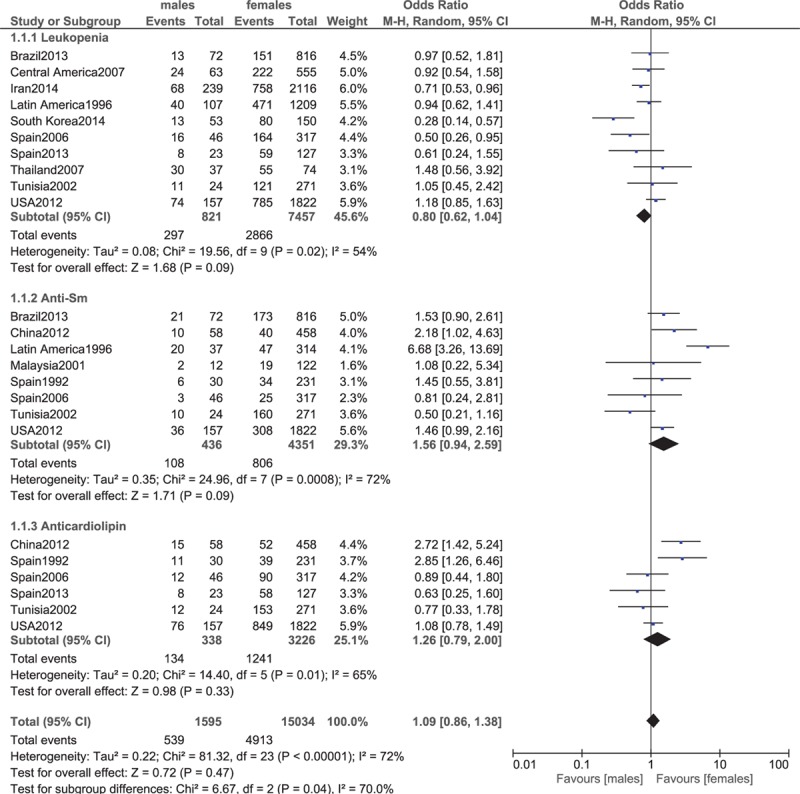
Leukopenia, anti-Sm antibodies, anticardiolipin antibodies.

**Figure 8:**
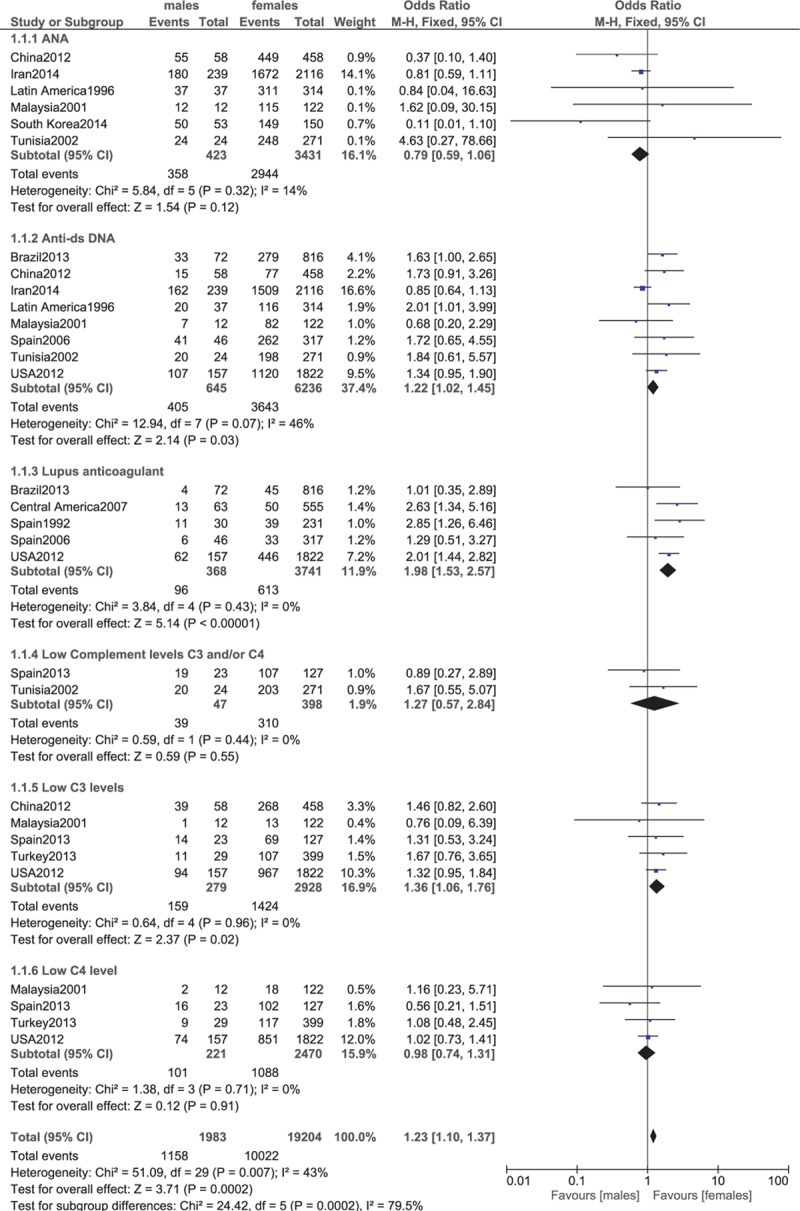
ANA, anti-dsDNA, lupus anticoagulant, low level of C3, low C4 level.

The average female-to-male ratio of all the included studies is around 9.3:1.The forest plots provided pooled OR estimates indicating which clinical features were more common in male patients versus female patients. Results have been summarized in Table 7. The differences in manifestations between male and female patients are shown in Figs. 2–8.

**Table 7:**
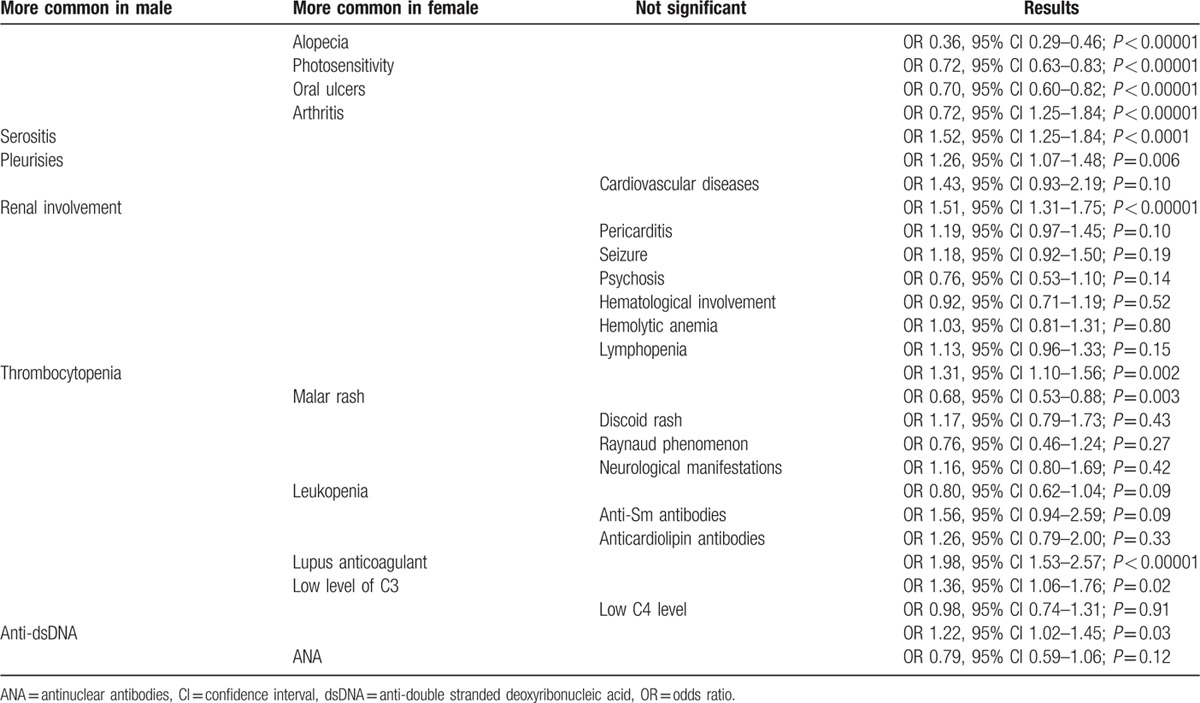
Comparison of clinical manifestations in male and female patients.

Our analysis, which compared the clinical features between males and females with lupus, showed that alopecia, photosensitivity, and oral ulcers were significantly higher in female patients (OR 0.36, 95% CI 0.29–0.46, *P* < 0.00001; OR 0.72, 95% CI 0.63–0.83, *P* < 0.00001; and OR 0.70, 95% CI 0.60–0.82, *P* < 0.00001, respectively). These results have been represented in Fig. 2.

Arthritis was also significantly lower in male patients (OR 0.72, 95% CI 1.25–1.84, *P* < 0.00001). However, serositis and pleurisies were significantly higher in male patients (OR 1.52, 95% CI 1.25–1.84, *P* < 0.0001; and OR 1.26, 95% CI 1.07–1.48, *P* = 0.006, respectively). Cardiovascular diseases favored females (OR 1.43, 95% CI 0.93–2.19, *P* = 0.10); however, the result was not statistically significant. These results have been represented in Fig. 3.

Our analysis showed renal involvement also to be significantly lower in female patients (OR 1.51, 95% CI 1.31–1.75, *P* < 0.00001). Pericarditis, seizure, and psychosis were almost similarly manifested between male and female patients with lupus (OR 1.19, 95% CI 0.97–1.45, *P* = 0.10; OR 1.18, 95% CI 0.92–1.50, *P* = 0.19; and OR 0.76, 95% CI 0.53–1.10, *P* = 0.14, respectively). These results have been represented in Fig. 4.

Hematological manifestations, as a whole, were similar between male and female patients with lupus (OR 0.92, 95% CI 0.71–1.19, *P* = 0.52). If analyzed individually, hemolytic anemia and lymphopenia were similar in males and females (OR 1.03, 95% CI 0.81–1.31, *P* = 0.80; and OR 1.13, 95% CI 0.96–1.33, *P* = 0.15, respectively). However, thrombocytopenia was significantly higher in male patients (OR 1.31, 95% CI 1.10–1.56, *P* = 0.002). These results have been represented in Fig. 5.

Since heterogeneity was higher while analyzing certain clinical features, a random-effect model has been used to analyze these features with high heterogeneity. Malar rash was significantly higher in female patients (OR 0.68, 95% CI 0.53–0.88, *P* = 0.003), whereas discoid rash was higher in male patients (OR 1.17, 95% CI 0.79–1.73, *P* = 0.43). However, the result for discoid rash was not statistically significant. Raynaud phenomenon and neurological manifestations were similar between males and females (OR 0.76, 95% CI 0.46–1.24, *P* = 0.27; and OR 1.16, 95% CI 0.80–1.69, *P* = 0.42, respectively). These results have been shown in Fig. 6.

Leukopenia was higher in female patients; however, the result was not statistically significant (OR 0.80, 95% CI 0.62–1.04, *P* = 0.09). Anti-Sm antibodies favored female patients (OR 1.56, 95% CI 0.94–2.59, *P* = 0.09. However, the result was not statistically significant in our study. Anticardiolipin antibodies were also similarly manifested between male and female patients (OR 1.26, 95% CI 0.79–2.00, *P* = 0.33). These results have been represented in Fig. 7.

Lupus anticoagulant was significantly higher in female patients (OR 1.98, 95% CI 1.53–2.57, *P* < 0.00001). Low level of C3 was also significantly apparent in females (OR 1.36, 95% CI 1.06–1.76, *P* = 0.02). Low C4 level was similarly observed in males and females (OR 0.98, 95% CI 0.74–1.31, *P* = 0.91). Anti-double stranded deoxyribonucleic acid (dsDNA) was significantly higher in male patients (OR 1.22, 95% CI 1.02–1.45, *P* = 0.03). Antinuclear antibodies (ANAs) favored male patients; however, the result was not statistically significant (OR 0.79, 95% CI 0.59–1.06, *P* = 0.12). These results have been represented in Fig. 8.

**Figure 9:**
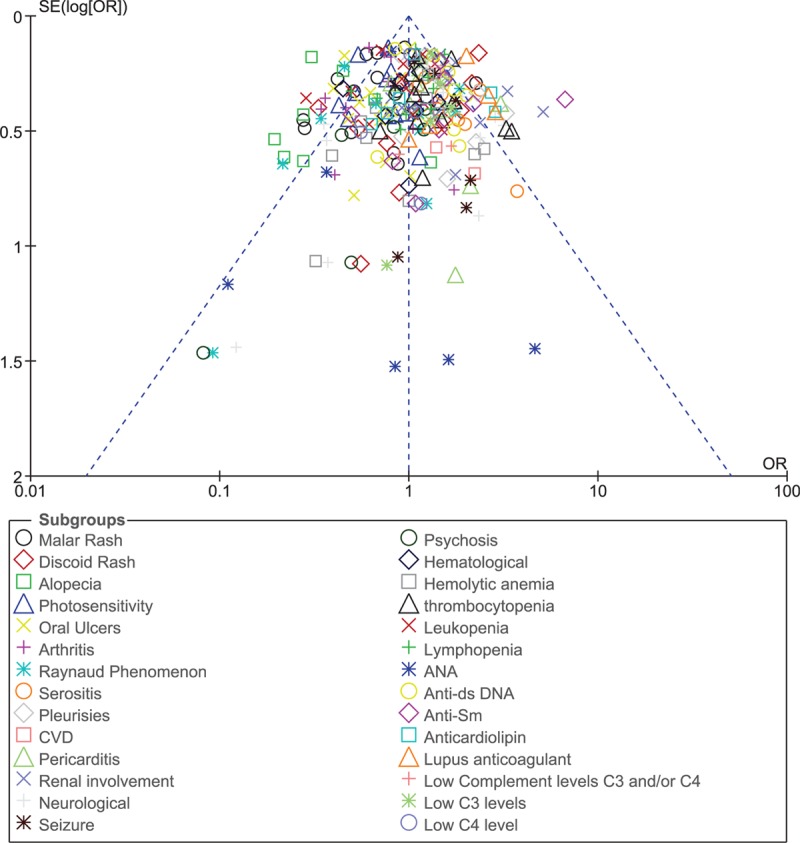
All clinical endpoints in male and female patients with lupus.

For all of the above analyses, sensitivity analyses yielded consistent results. Based on a visual inspection of the funnel plots, there has been no evidence of publication bias for the included studies that assessed all clinical endpoints in male and female patients with lupus. The funnel plot has been illustrated in Fig. 9.

## 4 Discussion

This study aimed to show the impact of sex on the clinical manifestations in SLE patients from different population groups. The mean average female-to-male ratio of all the included studies was 9.3:1. This reflects the results of most previous studies, which suggest female predominance in SLE.^[20,21]^ Several reasons have been brought forward to explain this. One of the main reasons is genetic susceptibility. At least 3 gene variants located on the X chromosome have been shown to be associated with increased risk of developing SLE (Interleukin-1 receptor-associated kinase 1, Methyl CpG binding protein 2, and toll-like receptor 7 [TLR7]). Another possible reason may be related to sex hormones.^[22]^ It is generally recognized that the male hormone, testosterone, is immunosuppressive, whereas the female hormone, estrogen, stimulates immune response.^[23,24]^ Lower testosterone levels have been observed in male and female patients with SLE. Several studies indicate that testosterone also interacts with the immune system by suppressing both cellular and humoral responses.^[25]^ Exacerbations of the disease activities of SLE are commonly noted during the premenstrual period, early pregnancy, and in the puerperium.^[26]^ This is suggestive of a close relationship between increasing concentrations of plasma estrogen and flare-ups of SLE.^[27]^ Estrogen seems to play an important role in promoting autoimmune-related immune responses, including the production of cytokines such as Th2 cytokines (e.g., interleukin [IL]-4, IL-6, and IL-10), antibodies, and endogenous autoantigens such as Human endogenous retroviruses (HERV).^[28–30]^ These HERV proteins seem to be related to autoantibody production, through molecular mimicry between HERV proteins and autoantigens such as ribonucleoprotein antigens, and are reported to be one of the pathogenic factors of SLE.^[30]^ Moreover, estrogens bind to and activate estrogen receptors which modulate the expression of many genes. The abnormal expression of estrogen or its receptors may lead to immunological diseases, including SLE. Possible mechanisms suggested for the high female predominance are fetal microchimerism, X chromosome inactivation, and X chromosome abnormalities.^[31]^ However, further research is warranted here. Specific mutations of X chromosome genes cause autoimmune syndromes characterized by different degrees of severity.^[32]^ Scofield et al suggested that the number of X chromosomes is another major cause of sex-specific difference because both the number of X chromosomes and genetic variants on the X chromosome are related to the risk of development of SLE. Hence, 2 functional X chromosomes, either by sex or by translocation or duplication, seem to confer a greater risk of SLE than 1 X chromosome.^[33]^ Male patients with Klinefelter syndrome (47,XXY) have similar risk to develop SLE compared with females (46,XX).^[34]^ It is also possible that women and men have different environmental exposures during their lifetimes, due to occupational or culturally-determined factors, which could be potentially linked to the increased incidence of SLE among women.

The mean age at disease onset and mean age at diagnosis of male and female patients in most of the included studies were comparable, as shown in Table 6. However, our data show a later age of disease onset and diagnosis in the studies from Spain.^[35,36]^ Several other European studies have reported peak incidences to occur at a later age in both European males and females.^[37–39]^ This has been attributed to genetic predisposition or the decreasing response of an aging immune system.^[40]^ Little research exists pertaining to the incidence or prevalence of SLE in many populations or their comprising ethnic groups. In the USA, the average incidence of SLE has been estimated to range between 1.8 and 7.6 cases per 100,000 person-years,^[41]^ and in Europe, the incidence rates range from 3.3 to 4.8 per 100,000 person-years.^[42]^ A study in Brazil detected an annual incidence of 8.4 per 100,000 habitants.^[43]^ The incidence of SLE is reported to be greater in Afro-Americans, Afro-Caribbeans, Native Americans, and Asians compared with Caucasians.^[44–46]^ In Taiwan, the incidence was reported to be 8.1per 100,000 persons in 2007.^[47]^ Geographic and environmental factors play an important role in the prevalence and general manifestations of SLE. Vilar and Sato^[43]^ described a high prevalence of cutaneous manifestations leading to a high incidence of the disease in Brazil due to the great amount of sunlight exposure. Genetic susceptibility interacts with lifestyle and environmental factors, which include socioeconomic status, infectious agents (triggering or protective agents), and environmental hazards in determining the risk of developing autoimmunity.

Although the included studies were from countries of different geographical locations with distinct environmental, sociocultural, economic and behavioral backgrounds, and unalike accessibility to health service facilities, they showed some similar outcomes when clinical features of males and females were compared. Serositis, pleurisies, and renal involvement were noted to be significantly higher in male lupus patients, whereas in female patients, arthritis and cutaneous manifestations such as malar rash, oral ulcers, alopecia, and photosensitivity were predominant in almost all of them. This is reflected in several other previous studies. Impaired renal function,^[48]^ renal failure,^[49,50]^ renal transplantation,^[51]^ chronic renal insufficiency,^[50]^ and renal end-stage disease^[52]^ were found to be more frequent in men than in women with SLE. Some series with biopsy results have shown a higher incidence of proliferative nephritis in males.^[53,54]^ Renal involvement in men is indicator of poor prognosis. It has been suggested that the main female hormone, 17β estradiol, is capable of inhibiting inflammatory and proapoptotic processes, and protecting the renal tissue, as opposed to the male hormones, testosterone and dehydroepiandrosterone.^[55]^ With respect to hematological and autoantibody profiles, the incidence of leukopenia, presence of lupus anticoagulant, low levels of C3, and positive titers of ANA were higher in females, whereas in males, thrombocytopenia and positive titers of anti-dsDNA were more prevalent. Scofiel et al suggested that men are more likely to have thrombocytopenia, which is associated with serositis, neuropsychiatric disease, renal disease, and positive dsDNA titer, and which is an indicator of a more severe disease in SLE. Thrombocytopenia has been linked to genetic predisposition.^[56]^ Some of the antibodies have been associated with specific manifestations of the disease; for example, anti-dsDNA and anti-Sm antibodies are associated with nephritis.^[57]^

### 4.1 Limitations

Several limitations are present in this current study. Firstly, variability in cohort sizes and lengths of follow-up may not bring uniformity among the included studies. Secondly, we have not elaborated on the sex-specific differences in each ethnic group of each study due to lack of data. Moreover, the specific differences in pathogenesis and target organ damage amongst sexes, which have only been explained partly though genetic, hormonal, and immune responses, have been analyzed.

## 5 Conclusions

This is a quantitative analysis of multiple studies comparing various clinical manifestations, autoantibodies, and laboratory results of male and female lupus patients. The results of this meta-analysis suggest that alopecia, photosensitivity, oral ulcers, arthritis, malar rash, lupus anticoagulant level, and low level of C3 were significantly higher in female lupus patients, whereas renal involvement, serositis and pleurisies, thrombocytopenia and anti-dsDNA level were predominant in male patients. However, more clinical and population-based research is warranted to further elucidate these differences and permit the development of optimal sex-tailored treatment and better outcomes for patients.
